# Laparoscopic Resection for Rectal Cancer: What Is the Evidence?

**DOI:** 10.1155/2014/347810

**Published:** 2014-04-16

**Authors:** Dedrick Kok-Hong Chan, Choon-Seng Chong, Bettina Lieske, Ker-Kan Tan

**Affiliations:** Division of Colorectal Surgery, University Surgical Cluster, National University Health System, 1E Kent Ridge Road, Singapore 119228

## Abstract

Laparoscopic colectomy for colon cancer is a well-established procedure supported by several well-conducted large-scale randomised controlled trials. Patients could now be conferred the benefits of the minimally invasive approach while retaining comparable oncologic outcomes to the open approach. However, the benefits of laparoscopic proctectomy for rectal cancer remained controversial. While the laparoscopic approach is more technically demanding, results from randomised controlled trials regarding long term oncologic outcomes are only beginning to be reported. The impacts of bladder and sexual functions following proctectomy are considerable and are important contributing factors to the patients' quality of life in the long-term. These issues present a delicate dilemma to the surgeon in his choice of operative approach in tackling rectal cancer. This is compounded further by the rapid proliferation of various laparoscopic techniques including the hand assisted, robotic assisted, and single port laparoscopy. This review article aims to draw on the significant studies which have been conducted to highlight the short- and long-term outcomes and evidence for laparoscopic resection for rectal cancer.

## 1. Introduction

The role of laparoscopic resection in the management of colon cancer is now widely accepted following several large-scale randomised controlled trials [1–6]. In terms of oncologic outcomes, these studies demonstrated comparable long-term results between the laparoscopic and open approaches. The rates of local recurrence, disease-free survival, and overall survival were not statistically different between the two groups [6–10]. In addition, the laparoscopic approach conferred reduced postoperative wound pain, decreased length of hospital stay, earlier return of bowel function, and improved cosmetic outcomes [1–3, 5–7].

Yet, surgery in rectal cancer poses a different set of challenges. The completeness of total mesorectal excision (TME) and a negative circumferential margin (CRM) are important prognostic factors. A deep and narrow pelvis in some patients and the effects of neoadjuvant chemoradiation treatment make the operation more demanding. Moreover, there is the need to minimise disruption to autonomic nerves that are responsible for postoperative urinary and sexual function [8].

The lack of well-conducted large-scale randomised studies on the benefits and implications comparing laparoscopic and open proctectomy for rectal cancer only posed questions on the role of laparoscopic resection in rectal cancer. Considerable differences in the anatomical definition of the rectum, exact location of the cancer, patient selection for neoadjuvant therapy, and the laparoscopic technique adopted among various authors impede any meaningful conclusions from studies published to date.

This review will highlight the short- and long-term outcomes comparing the open and laparoscopic approaches in the management of rectal cancers from an oncologic and functional perspective. In addition, comparisons between the various laparoscopic techniques available will be discussed.

## 2. Results

### 2.1. Short-Term Outcomes

Oncological outcomes in terms of the completeness of the TME, involvement of the circumferential resection margin (CRM), and number of lymph nodes harvested have key implications on long-term outcomes. Involvement of the CRM has been shown to increase rates of local recurrence considerably [9]. Better visualisation from clearer and sharper images and manoeuvrability within a tight pelvis with the laparoscope have all been cited as possible reasons for improved TME completeness [10–14]. As seen in Table 1, the short-term outcomes of the multicentre RCTs only demonstrated that laparoscopic proctectomy was only as good, but not better than open surgery, in terms of the aforementioned oncologic parameters.

From the 5 RCTs and a large-scale multicentre prospective review by Lujan et al., there were no statistical differences in the completeness of the TME, involvement of the circumferential resection margin (CRM), and number of lymph nodes harvested. In all the studies, whilst a formal pathological evaluation was undertaken, different standards were applied to each study and overall comparison was difficult to make. The COREAN trial evaluated completeness of TME via a grading specified by Nagtegaal et al. [15], in which the macroscopic qualities of the specimens were tagged. The COLOR II trial used a classification dependent on macroscopic quality as described by Quirke [16]. CRM was considered positive in the COREAN trial when distance from the tumour to the mesorectal fascia was less than or equal to 1 mm. The distance used in COLOR II was 2 mm. Only the two studies by Lujan et al. found that laparoscopic surgery resulted in an increase in TME completeness, reduced CRM positivity [17], and an increased lymph node yield [18].

The COREAN trial also needs to be specially mentioned because it was the only trial in which all patients enrolled had undergone neoadjuvant chemoradiotherapy. They highlighted that the laparoscopic approach was able to achieve comparable outcomes to open surgery in terms of surgical morbidity and mortality, as well as oncologic resection [19].

In all the studies, surgical times were significantly longer when the laparoscopic approach was adopted. Apart from two studies, the differences in the length of hospital stay between the two groups were actually not considerably different. In comparison with open surgery, the length of stay tended to be slightly shorter with the laparoscopic approach, albeit without statistical significance. Although intraoperative blood loss was found to be significantly less in the laparoscopic group, the safety of the laparoscopic approach in terms of perioperative morbidity and mortality has been shown to be equivalent to the open method.

### 2.2. Long-Term Outcomes

As seen in Table 2, only one multicentre and 2 smaller single-centre RCTs have been performed to address any differences in the long-term oncological outcomes between the 2 approaches [4, 18, 20]. With a median follow-up period of 3–5 years, these trials did not demonstrate any statistically significant difference in the long-term oncologic outcomes. There were no statistically different results in terms of local recurrence rates, disease-free survival, and overall survival. Results from the 2 smaller single-centre RCTs actually suggested that there was a tendency towards reduced local recurrence rates in the laparoscopic approach.

In spite of the above results, caution must be taken in their interpretation. The trial by Ng et al. did not include any patients who had neoadjuvant chemoradiation therapy due to the lack of evidence supporting its effectiveness during the earlier stages of the trial [20]. This trial also had relatively small numbers of patients in both arms. This made any meaningful analysis of oncologic outcomes more difficult. Although Lujan et al. recruited more patients to the trial, only a median follow-up duration of just under 3 years was reported [18]. A much longer duration of follow-up would have been preferred to confidently ascertain the oncologic outcomes of laparoscopic proctectomy compared to the open approach.

The only multicentre trial reported was the CLASICC trial. They recruited 326 patients with rectal cancer with a median follow-up period of 62.9 months. There was no statistical difference in the long-term outcomes between the 2 groups. More studies of this nature will need to be performed before conclusive evidence on the effectiveness of laparoscopic proctectomy can be made.

### 2.3. Functional Outcomes and Quality of Life

The preservation of both bladder and sexual function after surgery for rectal cancer has profound effects on quality of life. They should be considered in tandem with the oncological outcomes in a comparison between the open and laparoscopic approaches. In spite of efforts to identify and preserve nerves during open TME, the incidence of bladder and sexual dysfunction ranges from 0 to 12% and 10 to 35% of patients, respectively [21–24].

An analysis of the autonomic function was performed on eligible patients who were enrolled in the CLASICC, COLOR II, and COREAN trials [19, 22, 25].

Jayne et al. applied the International Prostatic Symptom Score (I-PSS) in the assessment of bladder function and the International Index of Erectile Function (IIEF) and Female Sexual Function Index (FSFI) for sexual function [22]. Scores were compared against the European Organization for Research and Treatment of Cancer QLQ-CR38 score which was collected during the CLASICC trial.

A global question which tested the patient's subjective interpretation of his overall condition revealed that equal proportions of patients following open and laparoscopic surgery reported bladder dysfunction. Specific symptom testing concurred with the above findings, with the most evident symptom affecting patients being a weak stream. There were 30% of patients in both the open and laparoscopic groups whom symptoms were characterized as being of moderate severity.

Amongst males, it was noted that 41% of patients reported a severe change in sexual function compared with 23% in the open rectal resection group. Although no significant differences in terms of symptom specific scores were found, there was a tendency for the laparoscopic approach group to experience erectile dysfunction at increased incidences. In the study, it was also noted that conversion to open surgery and performance of TME as opposed to wide mesorectal excision were significant predictors of poorer postoperative sexual function.

The survey actually yielded similar results among females. A total of 28% of patients in the laparoscopic resection group experienced overall decrease in sexual function “quite a lot” or “severely” compared to 17% in the open group. The main symptom reported was a dry vagina during intercourse.

Subgroup analysis of patients from the COLOR II trial by Andersson et al. did not just focus on bladder and sexual function but was more comprehensive in its analysis of overall quality of life [25]. The study administered the QLQ-CR38 instrument. Similar to Jayne et al., there was no difference in the QLQ-CR38 score between the open and laparoscopic groups. Prospective scores over time actually improved, albeit with no statistically significant difference. Sexual dysfunction was not analysed specifically but as part of general overall well-being in this study.

QLQ-CR38 was similarly evaluated in participants of the COREAN trial, and comparisons were made between bladder and sexual function at the preoperative phase and at 3 months after surgery. Micturition problems were found to be significantly less in patients who had undergone laparoscopic surgery. This was attributed by the authors to be due to the larger magnification and resultant better preservation of autonomic nerves. Male and female sexual problems were not found to be statistically different between the open and laparoscopic groups.

Formal subgroup analysis comparing the incidence of sexual and bladder dysfunction amongst laparoscopic technique in the above trials was not performed, though it has been noted in other smaller studies that laparoscopic abdominoperineal resections tend to have more dysfunction compared to laparoscopic anterior resection with TME [21]. Separately, Jones et al. noted that in their experience of 101 male patients who had undergone laparoscopic low anterior resection, ultralow anterior resection, and abdominoperineal resection, the incidence of bladder and sexual dysfunction was uncommon. No attempt at a calculation of statistical significance between the groups was attempted [26].

From the results of these studies, there appears to be no distinct advantage of the laparoscopic technique in preserving autonomic function compared to the open approach as previously purported. It must also be mentioned that the creation of stomas has a profound impact on quality of life. To our knowledge, there is no large-scale prospective study which directly measures the impact of laparoscopic or open proctectomy on stoma creation and its resultant impact on quality of life.

### 2.4. Comparisons between the Various Laparoscopic Techniques

Many new minimally invasive approaches have become commonplace in recent years. These include hand assisted laparoscopic surgery (HALS), robotic assisted surgery (RAS), single incision laparoscopic surgery (SILS), and natural orifice transluminal endoscopic surgery (NOTES).

HALS has the advantage of enabling tactile feedback to the surgeon, as well as allowing the surgeon's hand to assist with retraction, dissection, haemostasis, and organ removal [27, 28]. In an RCT involving 186 patients that compared HALS and the open approach in rectal cancer (Table 3), short-term outcomes in terms of complications were similar between both arms [29]. Notably, there was no conversion to the open technique in this study. Pathological findings in terms of number of lymph nodes harvested, distance of distal resection margin, and involvement of CRM were not statistically different between the two groups. The laparoscopic group conferred improved short-term outcomes such as reduced surgical blood loss, procedure time, recovery period, and hospital stay.

There was only one particular study that compared HALS to standard laparoscopic techniques in rectal cancer (Table 4) [30]. All patients in this study underwent ultralow anterior resection. The study showed that the standard laparoscopic approach took longer. This difference was actually statistically significant. Estimated blood lost, postoperative complications rate, and duration of hospital stay were similar between the standard and the hand assisted groups. There were also no statistical differences between number of lymph nodes resected, distal tumour margins, and involvement of CRM. These findings were similar to a single centre retrospective analysis of 129 rectal cancer cases, which assessed the feasibility of HALS as an approach for rectal cancer by reporting good short- and long-term outcomes including 42% of patients who achieved complete pathological response with only 4% with tumour recurrence at the 3-year period [31]. No statistical analysis was made in this retrospective study.

Another increasingly adopted technique is the robot assisted laparoscopic approach. Conventional laparoscopic surgery has been suggested as being limited in terms of 2-dimensional visualisation, reduced dexterity, inflexible surgical instruments, need of a skilled surgical assistant, surgeon tremor, and poor ergonomics [32–34]. Robotic surgery offers 3-dimensional and magnified view, surgical instruments that enable seven degrees of intracorporeal movement and is predominantly surgeon-led with minimal reliance on a skilled surgical assistant [35–38].

The ongoing ROLARR trial was developed explicitly to compare the oncological, functional outcomes and the cost effectiveness of robotic assisted surgery for rectal cancer [39]. It is the only large-scale multicentre randomised controlled trial that was targeted to address the effectiveness of robotic assisted laparoscopic surgery against conventional laparoscopic surgery for rectal cancer.

Smaller studies have described their experiences with the laparoscopic approach. The series by Scarpinata and Aly did not highlight any difference in CRM positivity in patients who underwent either the conventional or the robot assisted approach. This was in spite of the tumour being closer to the anal verge [32]. These findings were echoed in another paper by Erguner et al., which showed similar short-term outcomes between robot assisted laparoscopy and conventional surgery, with improved quality of resected specimen in terms of completeness of TME in the robotic group [40]. Robot assisted surgery has also been purported to be more advantageous in obese patients, in whom the standard laparoscopic approach tends to have a higher rate of conversion to the open technique [41]. Reasons cited for this reduction in conversion rate included better retraction, visualisation, and more precise dissection with the robot [42]. Similarly, preservation of the autonomic nerves, as well as preservation of bladder and sexual function, has also been cited as an advantage [43].

Three pronged studies have evaluated the various above-mentioned modalities. Kang et al. compared the open, standard laparoscopic and robotic assisted laparoscopic approaches (Table 5) [44]. In this retrospective study, case-matched analysis of patients who had undergone rectal resection for rectal tumours within 10 cm from the anal verge was performed. Perioperative outcomes showed that the minimally invasive techniques were able to confer significant advantages in terms of less intraoperative blood lost, as well as good pathological outcomes in terms of distance of distal margin, involvement of CRM, and number of lymph nodes harvested, albeit with significantly longer operating times. There was no statistical difference in terms of the overall complication rates although anastomotic leakage was actually higher in the laparoscopic group when compared to the open group. Wound infection and voiding problems were however higher in the open group.

Single incision laparoscopic surgery (SILS) is another novel approach to laparoscopic surgery. Studies done on this topic have generally been limited to retrospective case-control studies [45]. Kim et al. performed a review of cases performed for both colon and rectal cancers comparing the SILS and the conventional laparoscopic approach and found that SILS was both safe and feasible in comparison with conventional laparoscopy. It was also associated with shorter recovery times and length of hospital stay [46]. Although natural orifice transluminal endoscopic surgery (NOTES) deserves mention in any discussion on new techniques in laparoscopic surgery, the experience on this technique for cancer surgery has been largely confined to small studies [47].

## 3. Conclusion

Any technique for rectal cancer must not only ensure oncologically acceptable outcomes, but also a good standard in quality of life, including the preservation of bladder and sexual function. Whilst the role of the laparoscopic approach in colon cancer is well proven, whether this can be reproduced in surgery for rectal cancer still remains to be conclusively seen. The CLASICC trial remains the only multicentre RCT that evaluated the role of laparoscopic proctectomy in rectal cancer. The ongoing JCOG 0404, ACOSOG Z6051, and Australasian A La CaRT trials will shed further light on the effectiveness of the laparoscopic approach in rectal cancer eventually. There is also the need to ascertain the impact of the laparoscopic approach on functional outcomes. Meanwhile, while novel laparoscopic techniques, such as the robot, SILS and NOTES, continue to be developed and studied, there is the sense somewhat that the horse has been placed before the cart as the primacy of the laparoscopic technique over the open approach should be established first.

## Figures and Tables

**Table 1:** Short-term outcomes.

Trial	Type	Surgical method in comparison	Numbers	Mean/median lymph nodes harvested	CRM positivity	TME complete	Conversion to open (%)	Duration of intervention (min)	Blood loss (mL)	Length of hospital stay (days)	Morbidity within 28 days (%)	Mortality within 28 days (%)
CLASICC [3]	Multicentre RCT-UK	Open versus laparoscopic assisted	254 (O) versus 127 (L)	NA	14 versus 16 (NA)	NA	34%	135 versus 180 (no significance calculated)	NA	13 versus 11 (no significance calculated)	40 versus 37	NA

COREAN [19]	Multicentre RCT—South Korea	Open versus laparoscopic assisted	170 versus 170	18 versus 17	4.1 versus 2.9 (1 mm from margin)	74.7 versus 72.4	1.20%	197 versus 245*	217.5 versus 200*	9 versus 8	23.5 versus 21.2	0 versus 0

COLOR II [48]	Multicentre RCT—30 centres in 8 countries	Open versus laparoscopic assisted	364 versus 739	14 versus 13	10 versus 10 (2 mm from margin)	92 versus 88	17%	188 versus 240*	400 versus 200*	9 versus 8	37 versus 40	2 versus 1

Lujan et al. [17]	Multicentre non randomised-72 centres in Spain	Open versus laparoscopic assisted	3018 versus 1387	14.75 versus 14.53	16.3 versus 9.5 (1 mm from margin)*	75.6 versus 82.4	17.37%	186.38 versus 217.83*	NA	11 versus 8*	45.6 versus 38.3*	3.6 versus 1.2*

Lujan et al. [18]	Single centre RCT-Spain	Open versus laparoscopic assisted	103 versus 101	11.6 versus 13.6	2.9 versus 4.0 (1 mm from margin)	NA	7.90%	172.9 versus 193.7	234.2 versus 127.8	9 versus 8	33.0 versus 33.7	2.9 versus 1.9

Ng et al. [20]	Single centre RCT-HK	Open versus laparoscopic APR	48 versus 51	13 versus 12.4	4.2 versus 5.9 (NA)	NA	9.80%	163.7 versus 213.5	555.6 versus 321.7	11.5 versus 10.8	52.1 versus 42.1	2.8 versus 2.5

*P < 0.05.

**Table 2:** Long-term outcomes-oncological.

Trial	Type	Surgical method in comparison	Numbers	Median follow-up period (months)	Local recurrence	Neoadjuvant therapy administered	Disease-free survival (months)	Median overall survival (months)
CLASICC [3]	Multicentre RCT—UK	Open versus laparoscopic assisted	254 (O) versus 127 (L)	62.9 in both arms	NA	Not controlled	67.1 versus 70.8	65.8 versus 82.7

Lujan et al. [18]	Single centre RCT—Spain	Open versus laparoscopic assisted	103 versus 101	34.1, 32.8	5.3 versus 4.8	77 versus 73, no statistical significance	81.0 versus 84.8	75.3 versus 72.1

Ng et al. [20]	Single centre RCT—UK	Open versus laparoscopic assisted	142 versus 136	136.6, 124.5	9.3 versus 5.5	Neoadjuvant not included in trial	∗	+

*Trial calculated probability of being disease-free at 15 years, 71.4% versus 79%.

^+^Trial calculated probability of overall survival at 15 years, 51.4% versus 47.4%.

**Table 3:** Open versus HAL [28].

	Open	HALS
Incision length (cm)	17 ± 2*	6 ± 1*
Procedure time (min)	140 ± 20*	161 ± 35*
Surgical blood loss (mL)	380 ± 85*	310 ± 96*
Hospital stay (days)	15 ± 3*	12 ± 2*
Complications	9	5
Pathological		
Median lymph nodes resected	15	16
Median length of distal margin (cm)	2	2
Number of specimens with involved CRM	0	0

*P < 0.05.

**Table 4:** Comparison of HALS versus standard laparoscopic approach.

Surgical method	Trial	Numbers	Estimated blood loss (mL)	Operating time (min)	Hospitalisation stay	Complications (%)	Number of LN harvested	Distal tumour margin (cm)	CRM	Involved CRM
Standard laparoscopic	Tjandra et al. [30]	31	152.9	188.2	5.8	25.8	17.4	2.6	8.3	1

HALS	Tjandra et al. [30]	32	158.1	169.8	5.9	21.9	17.4	2.6	8.5	1

HALS	Pendlimari et al. [31]	129	250	204	5	3	∗	∗	∗	∗

*Pendlimari et al.'s [31] analysed results for both rectal and colon cancers. No subdivision was made between the two types and hence cannot be accurately reflected here.

**Table 5:** Open versus standard laparoscopic versus RAS [43].

	Open	Laparoscopic	RAS
Procedure time (min)	252.6*	277.8*	309.7*
Surgical blood loss (mL)	275.4*	140.1*	133*
Hospital stay (days)	16*	13.5*	10.8*
Complications (%)	24.8	27.9	20.6
Pathological			
Median lymph nodes resected	17.4	15.6	15
Median length of distal margin (cm)	2.2	2	1.9
Number of specimens with involved CRM	17	11	7

*P < 0.05.
